# “*Enterocytozoon bieneusi* and *Cryptosporidium*: a cross-sectional study conducted throughout Thailand”

**DOI:** 10.1186/s12879-019-4422-4

**Published:** 2019-09-14

**Authors:** Rapeepun Prasertbun, Hirotake Mori, Yaowalark Sukthana, Supaluk Popruk, Teera Kusolsuk, Katsuro Hagiwara, Aongart Mahittikorn

**Affiliations:** 1 Department of Protozoology, Faculty of Tropical Medicine, Mahidol University, Bangkok, Thailand; 2 Department of General Medicine, Faculty of Medicine, Juntendo University, Tokyo, Japan; 3 Department of Helminthology, Faculty of Tropical Medicine, Mahidol University, Bangkok, Thailand; 4 Department of Pathobiology, School of Veterinary Medicine, Rakuno Gakuen University, Hokkaido, Japan

**Keywords:** *Enterocytozoon*, *Cryptosporidium*, Thailand

## Abstract

**Background:**

*Enterocytozoon bieneusi* and *Cryptosporidium* spp. are prevalent zoonotic parasites associated with a high burden among children. To date only limited molecular epidemiological data on *E. bieneusi* and *Cryptosporidium* spp. in humans living in Thailand has been published.

**Methods:**

PCR-based tools were used to detect and characterize *E. bieneusi* and *Cryptosporidium* spp. The internal transcribed spacer (ITS) region of the rRNA gene was used to investigate *E. bieneusi*, and the small subunit (SSU) rRNA gene was used to investigate *Cryptosporidium* spp., and 697 fecal samples from villagers and school children in rural areas in Thailand were analyzed.

**Results:**

The infection rates were 2.15% (15/697) for *E. bieneusi* and 0.14% (1/697) for *Cryptosporidium* spp. The prevalence of *E. bieneusi* was significantly high in Loei province. Sequence analysis indicated that the *Cryptosporidium* isolate was *C. parvum*. Nine *E. bieneusi* genotypes were identified, EbpC, Peru12, TMH6, TMH3, TMH7, H, D, and two novel genotypes TMLH1 and TMLH2. *E. bieneusi* prevalence was significantly higher in male participants than in female participants, and in children aged 3–15 years than in participants aged > 15 years.

**Conclusions:**

The prevalence, genotypes, and zoonotic potential of *E. bieneusi* were found to vary significantly high even in one country. Transmission routes and key animal carriers of *E. bieneusi* may be associated with differences in hygiene, sanitation, and cultural behaviors. Further molecular studies including longitudinal studies will be required to unveil epidemiological characteristics of these opportunistic intestinal protozoa in all over the countries.

## Background

Opportunistic intestinal protozoa such as *Cryptosporidium* spp. and *Enterocytozoon bieneusi* can cause diarrhea in humans [1], and are associated with increased mortality and short survival in immunocompromised people, especially AIDS patients [2]. Recently, intestinal cases of infection with these parasites have reportedly increased in non-HIV-infected populations such as organ-transplant recipients, patients with malignant diseases, and diabetes patients [3]. Although they are considered opportunistic pathogens, several outbreaks have been reported both in immunocompetent humans and in domestic and wild animals [4–7]. There are published reports of *E. bieneusi* and *Cryptosporidium* infection in children considered HIV-seronegative and in healthy children indicating the presence of enteric carriage of infection in immunocompetent people in developing countries and other regions [8]. The pathogens therefore pose significant challenges to public health, especially in developing countries because of their high prevalence and the disease burden associated with infections. They have also been linked to impaired growth and cognitive function in children and immunocompromised individuals [9–11].

Among the 14 *Cryptosporidium* species known to infect humans, *C. hominis* and *C. parvum* are the most predominant [12, 13], and among 14 species of microsporidia, *E. bieneusi* infects humans the most frequently. Transmission routes include direct contact with infected people (anthroponotic transmission) and animals (zoonotic transmission), and consumption of contaminated food and water [14, 15]. Molecular-based techniques are used to investigate prevalence, characterize species, determine genotypes, and assess the zoonotic potential of these parasites [16]. The prevalence and zoonotic potential of intestinal protozoa can vary by country and by location within a country.

Only limited molecular epidemiological data on *Cryptosporidium* and *E. bieneusi* have been published in humans in Thailand. To date, no studies on the prevalence or subtyping of *Cryptosporidium* in the community have been published. Previous studies investigating the prevalence and genotype characteristics of *E. bieneusi* in limited parts of Thailand revealed divergent genotype characteristics, and zoonotic potential [17, 18]. The aim of the current study was to determine the prevalence and genotype/subtype characteristics of *Cryptosporidium* and *E. bieneusi* in the community throughout Thailand, and evaluate the divergence and zoonotic risks associated with the pathogens. We conducted a molecular survey among villagers and school children in northern, northeastern, central, western, and southern Thailand.

## Methods

### Study sites and fecal collection

**Fig. 1:**
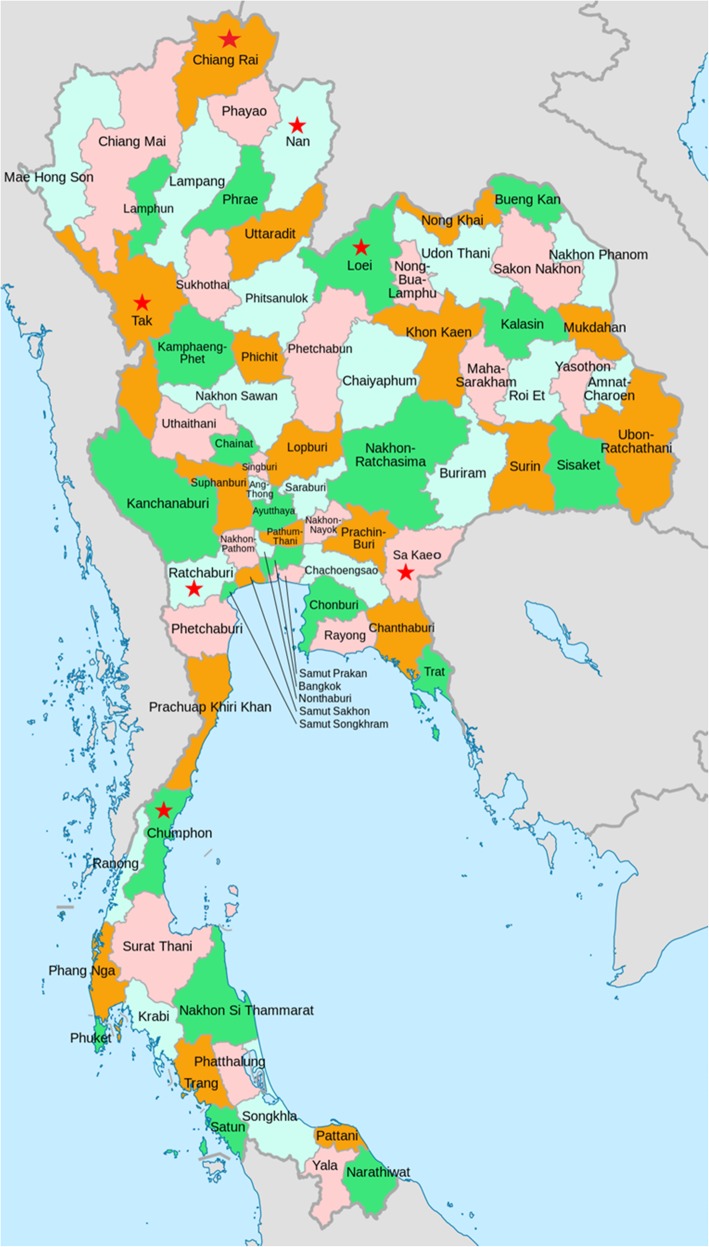
Map showing the geographic locations of the studied populations (modified from https://upload.wikimedia.org/wikipedia/commons/c/c5/Thailand_provinces_en.svg)

A total of 697 fresh fecal samples were collected from 235 healthy villagers and 462 healthy school children from August 2015 to January 2017 in rural areas in Thailand. Of the 697 participants, 318 were male and 379 were female. The samples were grouped into two age classes (3 to ≤15 years and >  15 years), and they were obtained from seven provinces; Chiang rai (*n* = 32) and Nan (*n* = 46) in the northern area, Tak (*n* = 151) and Ratchaburi (*n* = 166) in the western area, and Loei (*n* = 37), Chumphon (*n* = 101), and Sa kaeo (*n* = 164) respectively located in the northeastern, southern, and eastern areas (Fig. 1).

Compared with many other areas in Thailand, the study sites are rural and impoverished. The provision of infrastructure such as water supply, roads, and health services is not satisfactory. Most of the residents work in agriculture. In rural communities, major problems that have been identified are poverty, illiteracy and unemployment. Due to lack of literacy skills, inhabitants may experience problems with poor hygiene and sanitation conditions.

To preserve anonymity, each sample was given a unique identification number. All samples were collected from participants at home, kept at 4 °C, then transported to a laboratory at the Faculty of Tropical Medicine, Mahidol University, Thailand in ice packs. Fresh unpreserved fecal samples were aliquoted upon receipt and stored at − 20 °C prior to DNA extraction.

### Molecular analysis

DNA was extracted using the PSP Spin Stool DNA Kit (Stratec Inc., Germany) in accordance with the manufacturer’s instructions. The kit has a rigorous prelysis step using Zirconia Beads II with an optimized prelysis buffer under high temperatures that helps to extract DNA, followed by a preincubation of the sample with InviAdsorb to remove PCR inhibitors very efficiently. For *Cryptosporidium*, fragments of 18S rRNA (830 bp) were amplified via nested PCR as previously described [19]. PCRs were performed in a 25-μL reaction containing 1 × PCR buffer, 1.5 mM MgCl_2_, 0.2 mM of each dNTP, 2.5 U *Taq* polymerase (Thermo Scientific, USA), 1.0 μM of each primer, and 2.0 μL of DNA template. Each PCR consisted of thirty-five cycles of 94 °C for 45 s, 55 °C for 45 s, and 72 °C for 60 s. The primary PCR products were then used in second-round PCRs, which entailed thirty-five cycles of 94 °C for 45 s, 58 °C for 45 s, and 72 °C for 60 s.

For *E. bieneusi,* the internal transcribed spacer (ITS) region with a portion of the flanking large and small subunit (SSU) ribosomal RNA genes (390 bp) was amplified [20]. Negative and positive controls were included in all PCRs. Positive control samples were obtained using DNA polymerase known to test-positive and confirmed at the nucleotide level via GenBank, and nuclease-free water was used as a negative control. The PCR mixture was the same as that used for *Cryptosporidium* except that 2.0 mM MgCl_2_ was used. Both primary and second-round PCRs entailed thirty-five cycles (denaturation at 94 °C for 30 s, annealing at 55 °C for 30 s, and elongation at 72 °C for 40 s). Ten microliters of PCR products were subjected to electrophoresis in 1.5% agarose gel and visualized via ethidium bromide staining for 10 min in a 1 μg/mL ethidium bromide solution. The 100 bp DNA ladder (Thermo Scientific, USA) was included in every gel. PCR products were purified using spin columns (QiaQuick PCR purification kit, Qiagen, Germany) and sequenced in both directions using the secondary PCR primers for each organism and an ABI 3730xl DNA analyzer (Applied Biosystems, USA).

Genotypes/subtypes from each positive sample were confirmed by homology between the sequenced PCR products and published sequences in GenBank as determined via the Basic Local Alignment Search Tool (BLAST). The sequence of *E. bieneusi* novel genotypes TMLH1 and TMLH2 in the present study were submitted and deposited in GenBank with the respective accession numbers MG366589 and MG366590.

Phylogenetic analyses of *E. bieneusi* ITS sequences were performed using MEGA Software Version 7 [21]. Evolutionary distances between different isolates were calculated using the Kimura 2-parameter method [22], and phylogenetic trees were constructed using the neighbor-joining algorithm [23]. Branch reliability was assessed using bootstrap analyses (1000 replicates). The Chi-square test was performed using SPSS statistics 18 software (IBM, USA). *P* values < 0.05 were considered significant in all statistical analyses.

## Results

*Cryptosporidium* spp. was only detected in 1 sample in the entire study, from Tak Province, yielding a prevalence of 0.14% (1/697). The total prevalence of *E. bieneusi* was 2.15% (15/697). *E. bieneusi* was detected in samples from 3 provinces, Loei (northeastern area), Tak (western area), and Ratchaburi (western area). The prevalence of *E. bieneusi* was significantly higher in Loei (18.9%) than it was in Tak (2.6%) and Ratchaburi (2.4%). Prevalence of *E. bieneusi* varies significantly even in one country. *E. bieneusi* prevalence and genotype data are shown in Table 1.

**Table 1:** Age, sex distribution and genotype identification of *Enterocytozoon bieneusi* in fecal samples from human in rural areas, Thailand

Region	Province	Positive cases /total no. (%)					Genotype (number)
		Sex		Age		Total	
		Male	Female	3 to ≤15 Years	> 15 Years		
Northeastern	Loei	7/27 (25.9)	0/10 (0)	7/37 (18.9)	0/0 (0)	7/37 (18.9)*	TMH3 (1), TMH7 (2), TMH6 (1), H (1) ^***^, TMLH1 (1), TMLH2 (1)
Western	Tak	2/56 (3.6)	2/95 (2.1)	3/125 (2.4)	1/26 (3.8)	4/151 (2.6)	EbpC (1) ^***^, Peru12 (1) ^***^, D (2) ^***^
	Ratchaburi	4/104 (3.8)	0/62 (0)	4/166 (2.4)	0/0 (0)	4/166 (2.4)	EbpC (1) ^***^, TMH6 (2), TMH3 (1)
Northern	Chiang rai	0/18 (0)	0/14 (0)	0/9 (0)	0/23 (0)	0/32 (0)	
	Nan	0/14 (0)	0/32 (0)	0/3 (0)	0/43 (0)	0/46 (0)	
Southern	Chumphon	0/47 (0)	0/54 (0)	0/91 (0)	0/10 (0)	0/101 (0)	
Eastern	Sakaeo	0/52 (0)	0/112 (0)	0/31 (0)	0/133 (0)	0/164 (0)	
	Total	13/318 (4.1)**	2/379 (0.5)**	14/462 (3.0)**	1/235 (0.4)**	15/697 (2.15)	EbpC (2)^***^, D (2)^***^, TMH6 (3), TMH3 (2), TMH7 (2), H (1) ^***^, TMLH1 (1), TMLH2 (1), Peru12 (1) ^***^

*P < 0.05 was considered statistically significant positive (Chi-square test)

**P < 0.05 was considered statistically significant (Fisher test)

***Zoonotic genotype

The prevalence of *E. bieneusi* was significantly higher in school children aged 3–15 years (3.0%) than in participants aged > 15 years (0.4%) (*P* = 0.0258), and it was significantly higher in male participants (4.1%) than in female participants (0.5%) (*P* = 0.0013). In Loei, *E. bieneusi* was only detected in school children aged 3–15 years (18.9%; no *E. bieneusi* was detected in samples from people aged > 15 years), and all positive *E. bieneusi* samples were from males (Table 1).

*E. bieneusi* genotypes EbpC, Peru12, TMH6, TMH3, and D were identified in Tak and Ratchaburi. In Loei, genotypes TMH3, TMH7, TMH6, H, and two novel genotypes TMLH1 and TMLH2 were found. TMLH1 and TMLH2 both differed from genotype TMP11 by a single base (TMLH1 position 88 A → G, TMLH2 position 126 A → G) (Table 2). Genotype TMH6 was the most frequently identified (3/15) in this study. Zoonotic genotypes (EbpC, Peru12, D, and H) were identified in samples from Loei, Tak, and Ratchaburi. In Tak, zoonotic genotypes were more frequently identified, but in other provinces anthroponotic genotypes (TMH3, TMH7, and TMH6) were more prevalent (Table 1). The sequences of the single *Cryptosporidium-*positive sample had 100% homology with those of *C. parvum* (GenBank accession number AY204230).

**Table 2:** The variations in ITS region sequence of rRNA gene of *Enterocytozoon bieneusi* isolates from human in this study compared with ITS sequences of five known genotypes

Genotypes (no.)		Nucleotide at position						GenBank accession no.
		71	85	88	126	131	133	
Novel	TMLH1	C	G	A	G	T	G	MG366589
	TMLH2	C	G	G	A	T	G	MG366590
Known	TMP11	C	G	G	G	T	G	KU353447
	EbpC	C	G	G	G	C	A	MH745039
	PigHN-32	C	T	G	A	T	G	MF406105
	KIN-1	C	G	G	G	T	A	KY495647
	H	T	T	G	A	T	G	KP318000

**Fig. 2:**
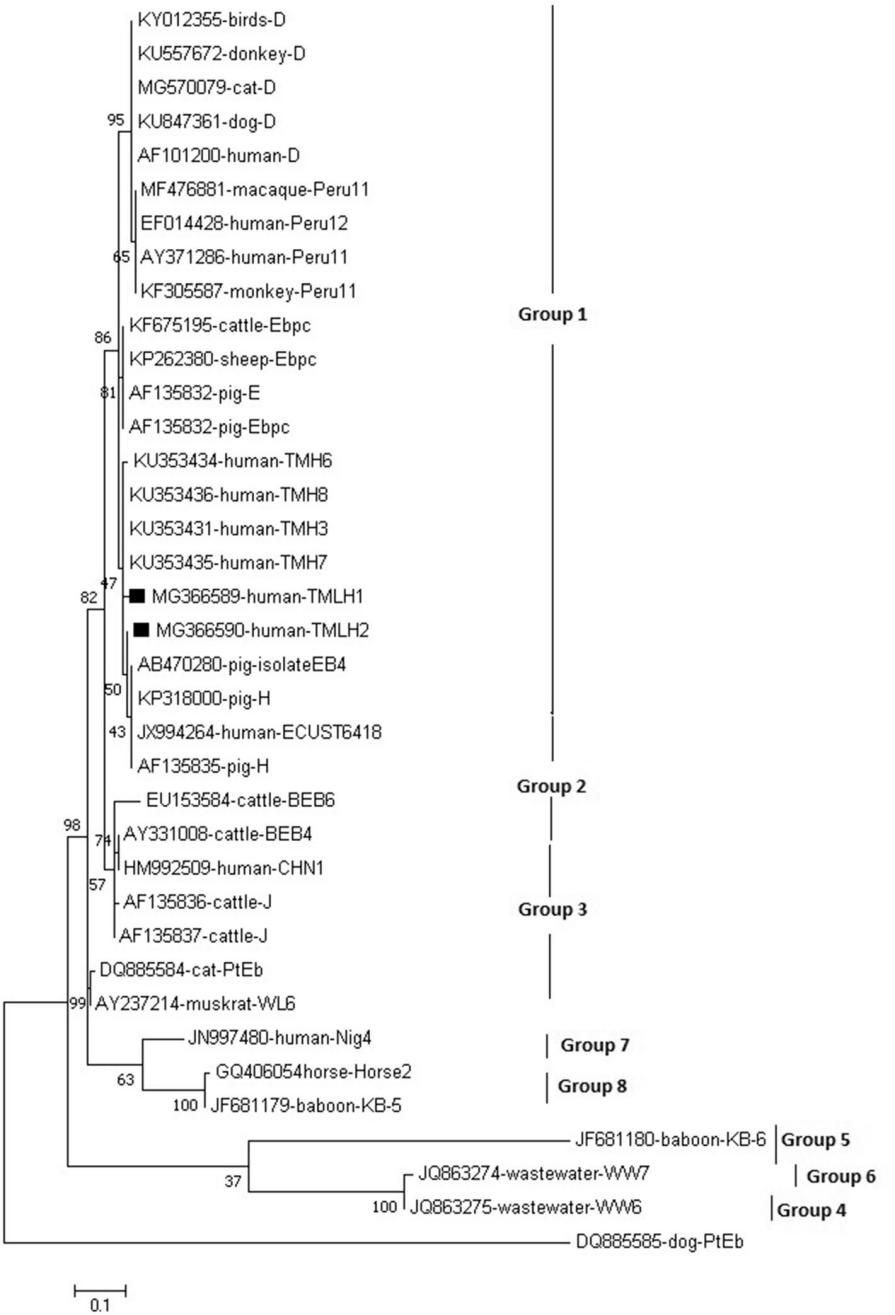
Phylogenetic analyses of *Enterocytozoon bieneusi* from human in the present study (15 cases positive) with GenBank accession number using Maximum Likelihood (ML) method in Mega 7.0 software (Gamma distributed with Invariant sites (G + I), 1000 replicates were performed. The two square filled in black indicates novel genotype identified in the present study which newly register to GenBank, three triangle indicates our genotype identified and publish previously [18]. These four circle indicates known genotypes identified in the present study, respectively. All of these genotype belong to group1 which is zoonotic group

In phylogenetic analysis, 13 ITS gene sequences were identical to seven genotypes and had 100% homology with those of genotypes D (GenBank accession number (AF101200), EbpC (KF675195), H (KP318000), TMH3 (KU353431), TMH7 (KU353435), TMH6 (KU353434), or Peru12 (EF014428). The remaining two were not previously described, and were designated TMLH1 (MG366589) and TMLH2 (MG366590). Phylogenetic analysis indicated that all of these genotype belong to group1 (Fig. 2).

## Discussion

The main objective of this study was to investigate the prevalence, genetic diversity, and zoonotic potential of *Cryptosporidium spp.* and *E. bieneusi* in humans in rural areas throughout Thailand. The prevalence of *Cryptosporidium* (0.14%) was lower than that previously reported in HIV-negative subjects in Thailand (1.0% [24], and in Honduras in children aged 0–5 years (56.4%; [25]. Overall, a range of 6 to 11% of water samples were contaminated with *Cryptosporidium* spp. and a occurrence of the parasite was reported in HIV/AIDS from 1996 to 2009 in Thailand at a range of 19 to 34% [26, 27]. The prevalence of *Cryptosporidium* varied in animal types. Infection rates between 5.7 and 31.5% have been reported in dairy cattle with dogs, cats and monkeys reflecting infection rates of 2.1, 2.5 and 1% respectively [28–31]. The distribution of *C. parvum* in humans varies according to different geographic areas and under different socioeconomic conditions. *Cryptosporidium* infection can be transmitted orally by drinking water and through environmental contaminants. Seasonality has also been reported to affect *Cryptosporidium* variation [32]. However, as our study spanned a period of 17 months, a seasonal effect is unlikely. The high prevalence of *Cryptosporidium* has been reported from water supplies in many parts of the world [4, 33, 34]. The low prevalence rate in our study may suggest an improved living standard in terms of environmental sanitary conditions shared by the study participants. Moreover, the water supply in Thailand is predominantly provided by the provincial waterworks authority which is unlikely to be the source of *Cryptosporidium*. In some European countries and New Zealand, both *C. hominis* and *C. parvum* are commonly detected in humans [35]. *C. parvum* is the predominant species detected in humans in developed countries such as England, France, and New Zealand, whereas *C. hominis* is reportedly the predominant species in developing countries [35]. In Thailand *C. hominis* (genotype 1), *C. meleagridis*, *C. muris*, and *C. felis* were reported in HIV-infected patients in 2002 [36]. In HIV/AIDS patients in Thai AIDS-care centers, *C. hominis* was the most commonly identified species, followed by *C. meleagridis* [26, 37]. The present study is the first molecular epidemiological investigation of *Cryptosporidium* in communities throughout Thailand, and the results suggest the epidemiological risks of *Cryptosporidium* may be minimal.

The prevalence of *E. bieneusi* determined in the present study was consistent with results of previous studies in human communities in Thailand. Respective prevalences of 3.8 and 2.9% have been determined in western and northern Thailand via nested PCR [17, 18]. However, it was reportedly more prevalent in children in China (22.5%; [8] and in the elderly in Spain (17.0%; [38]. In the current study prevalence varied by location. It was high in Loei in northeastern Thailand (18.9%), but the organism was not detected at all in four other provinces. Differences in reported prevalences may be associated with hygiene, sanitation, culture, living standards, methods of detection, and parameters used to define study populations.

Interestingly, in Loei and Ratchaburi *E. bieneusi* was more prevalent in males than females, which is concordant with a previous study in a remote city in the Brazilian Amazon [39]. It may be that the males in these studies engaged in agricultural and farming practices entailing a risk of contact with the parasite in the environment more frequently than the females. In addition, in the present study *E. bieneusi* was more prevalent in school children aged 3–15 years (3.0%) than in participants aged > 15 years (0.4%). A higher prevalence in children (19.0%) [40] than in adults (12.4%) has also been reported [41]. Similarly, in a study reported in 2002 that was conducted in a hospital in Uganda 17.4% of 1779 children with diarrhea were evidently infected with *E. bieneusi*, and it was concluded that *E. bieneusi* was widespread among children aged 3–36 months in Uganda [42]. Intestinal microsporidiosis may be more common in males than in females, and more common in children than in adults. Possible reasons for these findings could be as follows. First, personal hygiene and public health in agricultural and farming environments are poor when compared with other populations. Toilets are often not available in farm areas. Second, children of farming parents were more likely to be infected with intestinal parasites compared to children of parents who did not farm as these parents were more likely to have male children involved in casual labor [43]. Furthermore, male children are more likely to come in direct contact with the ground while playing [44]. Altogether, these can be related to anthroponotic transmission.

In the present study, genotype analysis of *E. bieneusi* also suggested zoonotic potential. The genotypes identified were EbpC, Peru12, D, H, TMH3, TMH6, TMH7, TMLH1, and TMLH2, and of these EbpC, Peru12, D, and H have been deemed indicative of zoonotic potential [18, 45, 46]. The genotypes identified in humans in Thailand to date include D, A, R, S, T, U, V, W, PigEb10, H, E, EbpA, O, Peru12, PigEbITS7, TMLH1–2, ETMK1 and TMH1–8 [17, 18, 37, 47–49] (Table 3).

**Table 3:** *E. bieneusi* genotype identified in humans and animals in Thailand

Genotype (synonym)	Hosts	References
D (WL8, CEbC, Peru9, PigEBITS9, PtEbV1)	Children, cat, human HIV+, pig	[17, 18, 37, 47], This study
A (Peru1, AF101197)	Human	[48]
R, S, T, U, V, W	Human HIV+	[47]
PigEb10	Human	[18]
H (PEbC)	Pig, Human	[18, 48], This study
EbpC (E)	Human, Pig	[17, 18, 47, 48], This study
EbpA (F)	Pig	[18]
O	Pig, Human	[18, 47, 48]
Peru12	Human	[47], This study
PigEbITS7	Pig	[47]
TMH1–8	Human	[18] This study
TMLH1–2	Human	This study
TMP1–11	Pig	[18]
ETMK1	Human	[17]
ETMK2–4	Cat	[49]

In Thailand genotype D is frequently found in humans and various other animal species [18, 47, 49], and genotypes EbpC, H, and Peru12 have also been reported [17, 37, 47, 48]. All are zoonotic genotypes. EbpC is reportedly a common genotype in non-human animals, especially in pigs and in China, where contact with pigs was evidently strongly associated with *E. bieneusi* genotype EbpC transmission to humans [50]. Genotype H has also been found in humans and pigs in China, Brazil, the Czech Republic, Germany, and Thailand [48, 51–54]. Genotype H was more commonly identified in humans and non-human primates in studies derived from China, Peru, Kenya, and Thailand [47, 55].

The detection of new *E. bieneusi* genotypes is common, and in the present study two novel genotypes were identified in humans in Loei. The novel genotype TMH2–8 was also detected in humans in Thailand in a previous study [18]. The frequent detection of new *E. bieneusi* genotypes in molecular epidemiological studies suggests genetic diversity of the species. In three provinces in which *E. bieneusi* was identified in the current study, anthroponotic genotypes were more prevalent than zoonotic genotypes in Loei and Ratchaburi, but in Tak the zoonotic genotypes D, EbpC, and Peru12 were predominant. Previous studies suggest that there is a substantial risk of zoonotic transmission in rural areas in Thailand [17, 18]. Human contact with other animals, especially pigs, may be a major contributor to the transmission of zoonotic genotypes to humans in Tak.

## Conclusions

In summary, in the present study in which fecal samples from 697 people residing in various geographically distinct rural areas in Thailand were analyzed *C. parvum* was the only *Cryptosporidium* species detected, and it was only detected in 1 person, representing a prevalence of 0.14%. This suggests that the risk of *Cryptosporidium* transmission to humans in rural areas in Thailand may be minimal. Conversely, the prevalence, genotypes, and zoonotic potential of *E. bieneusi* were found to vary significantly even in one country. Transmission routes and key animal carriers of *E. bieneusi* may be associated with differences in hygiene, sanitation, and cultural behavior. Further molecular studies, including longitudinal investigations, are required to more accurately characterize the epidemiological characteristics of these opportunistic intestinal protozoa.

## Data Availability

Representative sequences generated in this study were deposited in the GenBank database under the accession numbers MG366589-MG366590.

## Abbreviations

ITS: Internal transcribed spacer
SSU: Small subunit
